# The role of loneliness and negative schemas in the moment-to-moment dynamics between social anxiety and paranoia

**DOI:** 10.1038/s41598-023-47912-0

**Published:** 2023-11-26

**Authors:** Anson Kai Chun Chau, Suzanne Ho-wai So, Emma Barkus

**Affiliations:** 1 Department of Psychology, The Chinese University of Hong Kong, Hong Kong Special Administrative Region, New Territories, 3/F Wong Foo Yuan Building, Hong Kong, People’s Republic of China; 2 Institute of Health Equity, The Chinese University of Hong Kong, Hong Kong Special Administrative Region, Hong Kong, People’s Republic of China; 3 Department of Psychology, Northumbria University, Newcastle Upon Tyne, UK

**Keywords:** Psychology, Psychosis, Schizophrenia, Anxiety

## Abstract

Social anxiety and paranoia often co-occur and exacerbate each other. While loneliness and negative schemas contribute to the development of social anxiety and paranoia separately, their role in the development of the two symptoms co-occurring is rarely considered longitudinally. This study examined the moment-to-moment relationship between social anxiety and paranoia, as well as the effects of loneliness and negative schemas on both experiences individually and coincidingly. A total of 134 non-clinical young adults completed experience sampling assessments of momentary social anxiety, paranoia, and loneliness ten times per day for six consecutive days. Participants’ negative-self and -other schemas were assessed with the Brief Core Schema Scale. Dynamic structural equation modelling revealed a bidirectional relationship between social anxiety and paranoia across moments. Loneliness preceded increases in both symptoms in the next moment. Higher negative-self schema was associated with a stronger link from paranoia to social anxiety; whereas higher negative-other schema was associated with a stronger link from social anxiety to paranoia. Our findings support the reciprocal relationship between social anxiety and paranoia. While loneliness contributes to the development of social anxiety and paranoia, negative self and other schemas appear to modify the relationships between the two symptoms.

## Introduction

Around one-fifth of individuals with schizophrenia spectrum disorders have a comorbid social anxiety disorder (see meta-analysis by McEnery et al.^1^), characterized by an excessive fear or anxiety about scrutiny from others in social situations^2^. Compared with patients without comorbidity, those with comorbid social anxiety disorder report lower quality of life, more severe depression, and a higher rate of suicide^3–5^. The level of social anxiety in schizophrenia spectrum disorders needs only be at a subclinical level to be associated with poorer functioning, both concurrently and longitudinally^6^. This suggests that social anxiety may contribute to poorer outcomes in individuals with psychosis, even when present at subclinical levels.

Paranoia, the exaggerated belief that intentional harm is done or will be done by others^7^, is a common symptom of psychosis. Paranoia can manifest in milder forms as ideas of social reference or more severe forms as persecutory delusions ^8^. Albeit being distinct phenomena, paranoia and social anxiety are both characterized by appraisals of social threat: paranoia concerns imminent and ongoing physical, psychological or social harms by others^7^, whereas social anxiety reflects worry about rejection, embarrassment and scrutiny^9^. Among individuals with first-episode psychosis, those with comorbid social anxiety disorder reported more persecutory threats than those without comorbidity^3^. Across non-patient and community samples, correlations between subclinical levels of social anxiety and paranoia are consistently found to be moderate to strong^10–13^.

The co-occurrence of social anxiety and paranoia raises questions about how the two symptoms may influence each other^3,14^. On the one hand, it has been proposed that paranoia develops against the backdrop of anxiety and related worry processes^15–17^. The cognitive model of paranoia posits that social evaluative concerns, which form the core of social anxiety, contribute as an antecedent of paranoia^18^. In a longitudinal study with a community sample, Aunjitsakul et al.^19^ found that social anxiety at baseline predicted an increase in paranoia at 3-month follow-up. On the other hand, social anxiety has also been proposed to be a consequence of paranoid thinking, which inflicts internalized stigma and shame^20–22^. Two longitudinal cohort studies with general population samples^9,23^ found that paranoia at baseline predicted subsequent emergence of social anxiety, but not vice versa. However, these studies did not examine both directions of relationship in the same model. Therefore the covariation of the symptoms, which is conceptually interactive in nature, was not taken into full consideration.

Delineating the temporal dynamics between social anxiety and paranoia will not only reveal their potential bidirectional relationship, but also allow for investigations of putative underlying mechanisms. As both social anxiety and paranoia are experiences derived from the social environment, how connected one feels to the surrounding social environment could be important to the manifestation of these symptoms. Loneliness, a negative experience arising from a mismatch between perceived and actual social relationships^24^, has recently been implicated in the development of both social anxiety and paranoia^10,25–27^. It has been proposed that loneliness triggers heightened vigilance towards social threats, with an aim to protect the thwarted social relationships from further deterioration^24,28^. Heightened vigilance for social threats could be driven by an increase in negative affect^29^ or an attention bias towards cues of social threat in the external environment^30^, which may in turn precipitate heightened social threat appraisals. A 3-wave longitudinal study with a community sample^27^ found that loneliness predicted increases in social anxiety and paranoia, even after controlling for depression (which often co-exists with loneliness). This finding supports loneliness as a common psychopathological pathway to the development of both social anxiety and paranoia.

Apart from loneliness, negative schemas (i.e. global and stable beliefs about the self and others) may also underlie the development of social anxiety and paranoia. Cognitive models of social anxiety^18,31,32^ and paranoia^33,34^ have suggested that both symptoms build on negative-self beliefs (e.g., “I am worthless and weak”), which facilitate appraisals that one is vulnerable to social threats. Paranoia is suggested to be specific to negative-other beliefs (e.g., “Others are harsh and bad”)^10,35,36^, leading to a biased interpretation of others’ intention as hostile and malevolent. Recently, a cognitive model of social anxiety in schizophrenia highlights the role of negative-self schema^37^. The model posits that negative social situations activate preexisting negative self-representation, leading to the appraisal of social threats in the forms of social anxiety and paranoia. While the model does not specify the role of negative-other schema, we expect negative-other schema to contribute to a stronger tendency towards the formation of paranoia (relative to social anxiety).

As social anxiety, paranoia and loneliness occur naturalistically in the flow of daily life with varying intensities across hours and days, they can be reliably captured by the experience sampling method (ESM). ESM refers to repeated self-report questionnaires that record subjective experiences across moments in the flow of daily life^38^. Compared to traditional retrospective questionnaires, ESM represents these experiences with less recall bias^39^, which is particularly important when investigating momentary beliefs and appraisals. Importantly, ESM data provides valuable insights into the temporal dynamics between variables (i.e. cross-lagged effects) while taking into account their tendency to carry over across time (i.e. autoregressive effects)^40,41^. The autoregressive effects represent the extent to which a variable at the previous moment t-1 predicts itself at the current moment t, indicating the carry-over effect within the same variable across moments. On the other hand, the cross-lagged effects represent the extent to which a variable at the previous moment t-1 predicts change in another variable at the current moment t, indicating the spill-over effect from a variable to another variable across moments.

While previous studies mainly recruited samples across a large age span, the current study focused on young adulthood (i.e. age 18–30), a life stage where people are most vulnerable to loneliness, social anxiety and paranoia^42–44^. The aim of the present study was threefold: First, we tested the moment-to-moment dynamics between social anxiety and paranoia. We hypothesized significant cross-lagged effects from social anxiety to paranoia and vice versa. Second, we examined the moment-to-moment dynamics between loneliness and the two symptoms. We hypothesized significant cross-lagged effects from loneliness to both social anxiety and paranoia. Third, we tested the associations of core schemas with the strength of the cross-lagged effects. We hypothesized that negative-self schema would increase the strength of the cross-lagged bi-directional effects between social anxiety and paranoia. We also hypothesized a positive association between negative-other schema and the strength of the cross-lagged effect from social anxiety to paranoia.

## Results

### Sample characteristics

One hundred and fifty-four participants consented and took part in the study. Twenty participants were excluded due to a past or current psychiatric diagnosis (n = 18), or a subthreshold completion rate of the ESM assessment (see ‘Methods’ section, n = 2). Therefore, the final sample consisted of ESM data from 134 participants. The majority of our sample consisted of undergraduate students (n = 116, 86.6%), while the remaining (n = 18, 13.4%) were adults from the general population. A total of 5,800 ESM entries were entered into the Dynamic Structural Equation Modelling (DSEM) analyses (mean completion rate: 72.1%, SD = 0.16, range = 36.7% – 100%). The mean duration between two consecutive ESM entries within a day was 80.18 min (SD = 19.31, range = 15–147). The descriptive statistics of participants’ demographic information and baseline survey can be found in Table 1.

**Table 1:** Sample characteristics (N = 134).

	n (%)/M (SD)
Demographic characteristics	
Age	20.33 (2.91)
Gender	
Male	56 (41.8%)
Female	78 (58.2%)
Educational level attained	
Secondary education or below	104 (77.6%)
Associate degree or higher diploma	12 (9.0%)
Bachelor degree	11 (8.2%)
Master degree or above	7 (5.2%)
Employment status	
Full-time employment	12 (9.0%)
Part-time employment	81 (60.4%)
Not working	41 (30.6%)
Monthly household income	
< HKD 10,000	11 (8.2%)
HKD 10,000–29,999	38 (28.4%)
HKD 30,000–49,999	44 (32.8%)
HKD 50,000–99,999	27 (20.1%)
> HKD 100,000	14 (10.4%)
Key variables at baseline	
PHQ-9 total score	4.93 (4.31)
UCLA-LS-v3 total score	45.26 (10.56)
R-GPTS total score	9.91 (10.05)
SIAS-6/SPS-6 total score	12.00 (10.50)

*PHQ-9* the patient health questionnaire-9 scale; *UCLA-LS-v3* The University of California, Los Angeles, Loneliness Scale (Version 3); *R-GPTS* revised-Green et al. paranoid thinking scale; *SIAS-6/SPS-6* social interaction anxiety scale-6/social phobia scale-6.

### Dynamic structural equation modelling (DSEM) analyses

The estimates of the fixed and random effects in Models 1 and 2 are shown in Table 2. For Model 1, there were significant autoregressive effects for both social anxiety (β = 0.50, 95% CrI [0.28, 0.73]) and paranoia (β = 0.47, 95% CrI [0.24, 0.71]), indicating carry-over effects of social anxiety and paranoia across moments. The cross-lagged effect from social anxiety to paranoia (β = 0.20, 95% CrI [0.01, 0.41]) and the cross-lagged effect from paranoia to social anxiety (β = 0.29, 95% CrI [0.06, 0.51]) were both significant. We found no gender differences in the strength of these cross-lagged effects (*p* > 0.050).

**Table 2:** Fixed and random effects of the dynamic structural equation models.

Parameters	Model 1 (social anxiety and paranoia)				Model 2 (social anxiety, paranoia, and loneliness)			
	Within-person standardized fixed effects		Random effects		Within-person standardized fixed effects		Random effects	
	Estimate (β)	95% CrI	Estimate (variance)	95% CrI	Estimate (β)	95% CrI	Estimate (variance)	95% CrI
Intercepts/means								
μ_SA_	**2.08**	**[1.77, 2.41]**	0.80	[0.62, 1.05]	**1.94**	**[1.62, 2.27]**	0.95	[0.72, 1.29]
μ_PAR_	**2.02**	**[1.71, 2.33]**	0.62	[0.48, 0.81]	**1.88**	**[1.57, 2.20]**	0.72	[0.55, 0.98]
μ_LONE_	**/**	/	/	/	**1.96**	**[1.64, 2.28]**	0.84	[0.64, 1.29]
Autoregressive effects								
ϕ_SA⟶SA_	**0.50**	**[0.28, 0.73]**	0.15	[0.11, 0.21]	**0.41**	**[0.20, 0.63]**	0.20	[0.14, 0.28]
ϕ_PAR⟶PAR_	**0.47**	**[0.24, 0.71]**	0.14	[0.10, 0.20]	**0.31**	**[0.11, 0.52]**	0.18	[0.13, 0.26]
ϕ_LONE⟶LONE_	**/**	/	/	/	**0.61**	**[0.26, 0.86]**	0.11	[0.08, 0.16]
Cross-lagged effects								
ϕ_SA⟶PAR_	**0.20**	**[0.01, 0.41]**	0.10	[0.08, 0.14]	0.19	[− 0.01, 0.40]	0.11	[0.07, 0.15]
ϕ_PAR⟶SA_	**0.29**	**[0.06, 0.51]**	0.44	[0.32, 0.61]	**0.25**	**[0.04, 0.46]**	0.50	[0.35, 0.72]
ϕ_LONE⟶SA_	**/**	/	**/**	/	**0.26**	**[0.00, 0.52]**	0.08	[0.05, 0.12]
ϕ_SA⟶LONE_	**/**	/	**/**	/	0.05	[− 0.17, 0.27]	0.11	[0.07, 0.17]
ϕ_LONE⟶PAR_	**/**	/	**/**	/	**0.21**	**[0.01, 0.42]**	0.08	[0.05, 0.11]
ϕ_PAR⟶LONE_	**/**	/	**/**	/	0.19	[− 0.02, 0.40]	0.38	[0.27, 0.55]

*SA* social anxiety, *PAR* paranoia, *LONE* loneliness.

Significant estimates of the fixed effects based on the 95% credible interval (CrI) are in bold typeface.

See the notation of the parameters in Models 1 and 2.

For Model 1, the averaged within-person R^2^ for social anxiety and paranoia were 0.24 and 0.25, respectively. The averaged within-person R^2^ for social anxiety, paranoia and loneliness were 0.29, 0.28 and 0.24, respectively.

For Model 2, as shown in Table 2, the autoregressive effects of social anxiety (β = 0.41, 95% CrI [0.20–0.63]), paranoia (β = 0.31, 95% CrI [0.11, 0.52]) and loneliness (β = 0.61, 95% CrI [0.26–0.86]) were significant. The cross-lagged effects from loneliness to social anxiety (β = 0.26, 95% CrI [0.00, 0.52]) and paranoia (β = 0.21, 95% CrI [0.01, 0.42]) were both significant. There was also a significant cross-lagged effect from paranoia to social anxiety (β = 0.25, 95% CrI [0.04, 0.46]), but not vice versa (β = 0.19, 95% CrI [− 0.01, 0.40]). There were no gender differences in the strength of these cross-lagged effects (*p* > 0.050).

The between-person correlations between negative schemas and DSEM parameters for Model 2 are reported in Table 3. The level of negative-self was positively associated with the strength of the cross-lagged effect from paranoia to social anxiety (r = 0.32, 95% CrI [0.11, 0.51]). The level of negative-other schema was positively associated with the strength of the cross-lagged effect from social anxiety to paranoia (r = 0.30, 95% CrI [0.11, 0.49]), but negatively associated with the strength of the cross-lagged effect from loneliness to paranoia (r =  − 0.23, 95% CrI [− 0.43, − 0.02]). Both levels of negative-self (rs = 0.35–0.45) and -other schemas (rs = 0.23–0.29) were associated with mean social anxiety, paranoia and loneliness.

**Table 3:** Between-person correlations between negative schemas and random effects (Model 2).

Parameters	Negative-self schema	Negative-other schema
	r [95% CrI]	
Intercepts/means		
μ_SA_	**0.45 [0.27, 0.59]**	**0.29 [0.09, 0.46]**
μ_PAR_	**0.35 [0.17, 0.52]**	**0.23 [0.03, 0.41]**
μ_LONE_	**0.41 [0.23, 0.57]**	**0.27 [0.06, 0.45]**
Autoregressive effects		
ϕ_SA⟶SA_	− 0.08 [− 0.30, 0.14]	**0.22 [0.01, 0.41]**
ϕ_PAR⟶PAR_	**0.28 [0.06, 0.47]**	0.10 [− 0.12, 0.30]
ϕ_LONE⟶LONE_	**0.27 [0.05, 0.47]**	0.20 [− 0.03, 0.41]
Cross-lagged effects		
ϕ_SA⟶PAR_	− 0.04 [− 0.26, 0.18]	**0.30 [0.11, 0.49]**
ϕ_PAR⟶SA_	**0.32 [0.11, 0.51]**	0.10 [− 0.12, 0.31]
ϕ_LONE⟶SA_	− 0.06 [− 0.29, 0.20]	− 0.10 [− 0.33, 0.15]
ϕ_SA⟶LONE_	− 0.24 [− 0.46, 0.01]	0.03 [-0.21, 0.26]
ϕ_LONE⟶PAR_	− 0.05 [− 0.27, 0.18]	** − 0.23 [− 0.43, − 0.02]**
ϕ_PAR⟶LONE_	0.24 [− 0.00, 0.45]	0.15 [− 0.09, 0.35]

*SA* social anxiety, *PAR* paranoia, *LONE* loneliness.

Significant correlations based on the 95% credible interval are in bold typeface.

See the notation of the parameters in Model 2.

## Discussion

This study utilized ESM to repeatedly assess momentary symptoms in daily life and found that social anxiety predicted an increase in paranoia across moments and vice versa. Such reciprocal relationships were demonstrated in a sample of young adults in the absence of full-blown psychiatric disorders. These relationships did not differ between genders. Our findings showed that social anxiety and paranoia do not merely co-exist, but also dynamically interact with one another in their development and maintenance.

In addition to previous longitudinal studies which considered social anxiety and paranoia separately^9,19,23^, our analytical approach using DSEM focused on the covariation of both symptoms in a single model (Model 1). For the first time, we offered evidence for the bidirectional relationship within the same sample, revealing comparable effect sizes of each directional path. In addition to previous conceptualization of social anxiety as an antecedent to paranoia (e.g. cognitive model of paranoia, Freeman et al.^18^), our results also supported it as a consequence of paranoia as shown in other studies^9,23^. Future studies may clarify the overlap of paranoid thinking with the affective, cognitive and behavioral manifestations of social anxiety, which would inform the underlying processes in both symptoms.

We then took a closer look at loneliness in the moment-to-moment dynamics between social anxiety and paranoia (Model 2). We found that loneliness predicted an increase in both social anxiety and paranoia, corroborating with a longitudinal study with a community sample^27^. We confirmed the ‘healthy’ status of our sample with a psychiatric interview; therefore, our findings reflected the relationship between social anxiety and paranoia free from the confounding effects by treatments and chronicity of the psychiatric disorders. The convergent finding from Lim et al.^27^ and our study support loneliness as a common psychopathological pathway to both social anxiety and paranoia. While previous studies have found that loneliness leads to heightened vigilance for social threat via a myriad of affective and social-cognitive processes^29,30^, the contributions of these processes in differentiating social anxiety from paranoia outcomes need to be ascertained in further studies.

As hypothesized, the level of negative-self schema was associated with a stronger relationship from paranoia to social anxiety, whereas the level of negative-other schema was associated with a stronger relationship from social anxiety to paranoia. These findings were in line with the proposed role of negative beliefs about self (e.g. ‘I am worthless and weak’) in the formation of fear of rejection and criticism implicated in social anxiety^31,32^. The findings also supported the specificity between negative-other schema and paranoia^10,35,36^, where negative beliefs about others (e.g. ‘Others are harsh and bad’) would exacerbate the formation of paranoid thinking, possibly against the backdrop of social anxiety. Importantly, our findings highlighted the presence of both negative-self and -other schemas to be necessary to the maintenance of the reciprocal relationship between social anxiety and paranoia. This speculation is consistent with the finding of a recent latent profile analysis by Chau et al.^10^. They identified a subgroup of non-clinical young adults high on both social anxiety and paranoia, who reported more negative-self and -other schemas than subgroups high on either symptom. Future studies may examine how various constellations of negative-self- and -other schemas would shape the development of various phenotypic expressions of social anxiety and paranoia. Our findings also pave ways for future investigation of the potential between-person heterogeneity in these moment-to-moment dynamics, which may longitudinally predict the transition into social anxiety disorder, schizophrenia and their co-morbidity.

In sum, our findings offered support to Aunjitsakul et al.^37^’s unified framework for the understanding the psychopathological processes underlying social anxiety and paranoia. In particular, loneliness appears to be a situational trigger to the emergence of social anxiety and paranoia, in which their dynamics are strengthened by negative schemas. Our findings further extended Aunjitsakul et al.’s^37^ cognitive model with the role of negative-other schemas, which may exaggerate the appraisal of social threat in terms of harm and malevolence, which define paranoia^35^. Our findings shed light on the possibility of ameliorating social anxiety and paranoia via interventions that reduce loneliness^45,46^ or challenge negative-self and -other schemas (e.g. cognitive restructuring^47–49^).

There are several limitations of the current study. First, our results may be specific to the current sampling frequency of ESM assessment. Despite the statistical adjustment to confine the temporal effects to one-hour windows, it is inevitable that any effects that operate at shorter or longer time windows would be missed. Second, our data collection was conducted during the COVID-19 pandemic, a period when exacerbated loneliness, social anxiety and paranoia were reported^50–52^. Although the baseline levels of these phenomena were comparable to another sample of demographically diverse non-clinical young adults tested before the outbreak of the pandemic^10^ (N = 2089), we could not ascertain the confounding impact of the pandemic on the expression of these phenomena in daily life^36^. Third, a majority of our sample were undergraduate students. It is not sure whether our results would be replicated in demographically diverse samples. Finally, we acknowledge the possibility that the dynamics between social anxiety and paranoia may also involve other unmeasured mechanisms beyond loneliness and negative core schemas, such as interpersonal trauma^53,54^. This should be investigated in future research.

Using ESM, the current findings supported the reciprocal relationship between social anxiety and paranoia. Loneliness was also found to predict increases in both anxiety and paranoia across moments, suggesting that loneliness predates and may lead to the increases in both symptoms. Moreover, the strength of the dynamics between social anxiety and paranoia was associated with levels of negative-self and -other schemas. Our findings shed new light on the understanding of the dynamics between social anxiety and paranoia, which may invite replications in the clinical populations.

## Methods

Ethics approval for the study was granted by the Survey and Behavioral Research Ethics Committee of The Chinese University of Hong Kong (Reference no.: SBRE-19–788). All methods were carried out in accordance with relevant guidelines and regulations. Informed consent was obtained from all participants.

### Participants

Eligible participants aged 18–30 were recruited either from the subject pool of the Introductory Psychology course or via campus recruitment. Participants with any past or current psychiatric diagnosis (self-reported and then confirmed with a diagnostic clinical interview, see Measures) and who could not read Chinese were excluded. We targeted a sample size of 130, which is comparable to previous ESM studies with non-clinical samples analyzed using the dynamics structural equational modelling (DSEM) (see Statistical Analysis)^55–57^. Our targeted sample size fulfilled the sample size recommendation from a recent simulation study for DSEM^58^.

### Procedure

Data collection took place in June to October 2021. It happened to be after the peak of the fourth wave of the COVID-19 pandemic in Hong Kong. While face-to-face data collection was allowed by the university, territory-wide infection control measures such as social distancing and mask-wearing mandate were in place. Consented participants attended a 1-h assessment session during which they were screened with the Structured Clinical Interview for Diagnostic and Statistical Manual of Mental Disorders-IV (SCI-DSM-IV; So et al.^59^). Participants without any past or current psychiatric diagnosis completed a baseline survey, and were then briefed individually on the ESM procedure.

The ESM questionnaires were programmed into a smartphone app (SEMA3^60^) installed on the participant’s mobile phone. Adopting a signal-contingent sampling design, the app prompted participants to answer the same set of items assessing momentary loneliness, social anxiety and paranoia (see Measures below) ten times a day for six consecutive days. The app displayed the items one by one in a way that the preceding item had to be answered before the next item would appear. The prompt signals were pseudo-randomized into blocks of time intervals within 13 waking hours. The starting time of the ESM assessment was tailored for each participant to maximize compliance. Consecutive ESM questionnaires were set at least 15 min apart. Each ESM questionnaire expired in 15 min. The participant completed at least one ESM questionnaire as practice under the guidance of a research worker.

Support was rendered to the participants by the research team throughout the ESM assessment period. On the first assessment day, a research worker contacted the participant to ensure that the app was functioning properly and to encourage them to answer to the ESM prompts. In the middle of the week, the research worker monitored the participant’s progress and offered help to increase their compliance when necessary. Participants could also contact the research team whenever they encountered any difficulties with the app. After completing the 6-day ESM assessment, participants received course credits or monetary compensation for their time.

### Measures

#### Baseline survey

Participants completed retrospective questionnaires assessing levels of loneliness, paranoia, depression, and social anxiety. These included the UCLA-Loneliness Scale version 3 (UCLA-LS-v3)^61^, the Revised Green et al. Paranoid Thoughts Scale (R-GPTS)^62^, the Patient Health Questionannire-9 (PHQ-9)^63^ and the Social Interaction Anxiety Scale-6/Social Phobia Scale-6 (SIAS-6/SPS-6)^64^. The UCLA-LS-v3 and SIAS-6/SPS-6 do not specify the timeframe of reference, whereas PHQ-9 and R-GPTS assess depressive symptoms and paranoia within two weeks and one month respectively. The Chinese versions of these measures have been validated and used in previous studies^10,26,36^. Negative-self and -other schemas were measured with the respective subscales of the Brief Core Schema Scale^65^. Its Chinese version has been used in Chau et al.^10^ and So et al.^36^. Internal consistencies of these measures ranged from 0.78 to 0.93 in this sample. Items on age, gender, educational attainment, monthly household income and employment status were also included.

#### ESM assessment

All ESM measures were rated on a 7-point Likert scale (1 “not at all”–7 “very”).

##### Momentary loneliness

The 3-item UCLA Loneliness scale^66^ was modified to assess momentary loneliness (e.g., ‘I lack companionship right now’). These three items have been used in a previous ESM study^29^. In the current study, the within- and between-person reliabilities were 0.86 and 0.98 respectively.

##### Momentary social anxiety

Momentary social anxiety was assessed with the three items suggested in Kashdan and Steger^67^ (e.g., ‘I worried that I would say or do something wrong right now’). It has been used in previous ESM studies ^68,69^. In the current study, the within- and between-person reliabilities were 0.84 and 0.99 respectively.

##### Momentary paranoia

Momentary paranoia was assessed with the five items suggested by Schlier et al.^70^ (e.g., ‘People are trying to upset me right now’). These items have been used in previous ESM studies^70–72^. The within-(0.84) and between-person (0.99) reliabilities were good in the current study.

### Statistical analysis

In accordance with previous ESM studies, responses from participants who completed less than one-third of the total ESM questionnaires (i.e. 20) were excluded from the data analysis^73^. Our hypotheses were tested with Dynamic Structural Equation Modeling (DSEM)^40,41^. DSEM allows the examination of multi-level relationships among ESM variables by decomposing the intensive longitudinal data into within- and between-person variance components using a latent person-mean approach. For the within-person components, the fixed effects of means of ESM variables (i.e. intercepts), their autoregressive effects and cross-lagged effects were simultaneously estimated in a single model. The autoregressive effects were estimated by regressing the variables at the current moment t on the same variables at the previous moment t-1, while the cross-lagged effects were estimated by regressing the variable at the current moment t on another variable at the previous moment t-1. To allow for inter-individual differences in these fixed effects, the DSEM estimated all the random effects at the between-person level, which were allowed to correlate with each other.

Bayesian estimation is supported in DSEM to estimate all random effects in a single model with high accuracy and computational efficiency. The default non-informative priors were used in this study. Four Markov Chain Monte Carlo (MCMC) chains with 5000 iterations each were used, with a thinning of 10. Missing data was assumed to be missing at random and handled with MCMC sampling^40^. Within-person standardized parameters of the fixed effects^74^ were computed for interpretation. Estimates of all fixed effects were regarded as statistically significant if their 95% credible intervals (CrIs) did not include zero. Tests for model comparison were not conducted, as model comparison is an underdeveloped area for DSEM^40^.

To control for potential trends or non-stationarity of ESM data, the hour of measurement was added in the within-person level of the DSEM models as fixed effects. Unequal time spacing of the ESM data was handled by creating time grids of one hour using a discrete time filter approach using the Mplus option TINTERVAL^40^. Therefore, the interpretation of all parameters was in reference to the time window of one hour. A simulation study indicated that estimates of parameters are unbiased up to 80–85% of missing data^58^.

**Figure 1:**
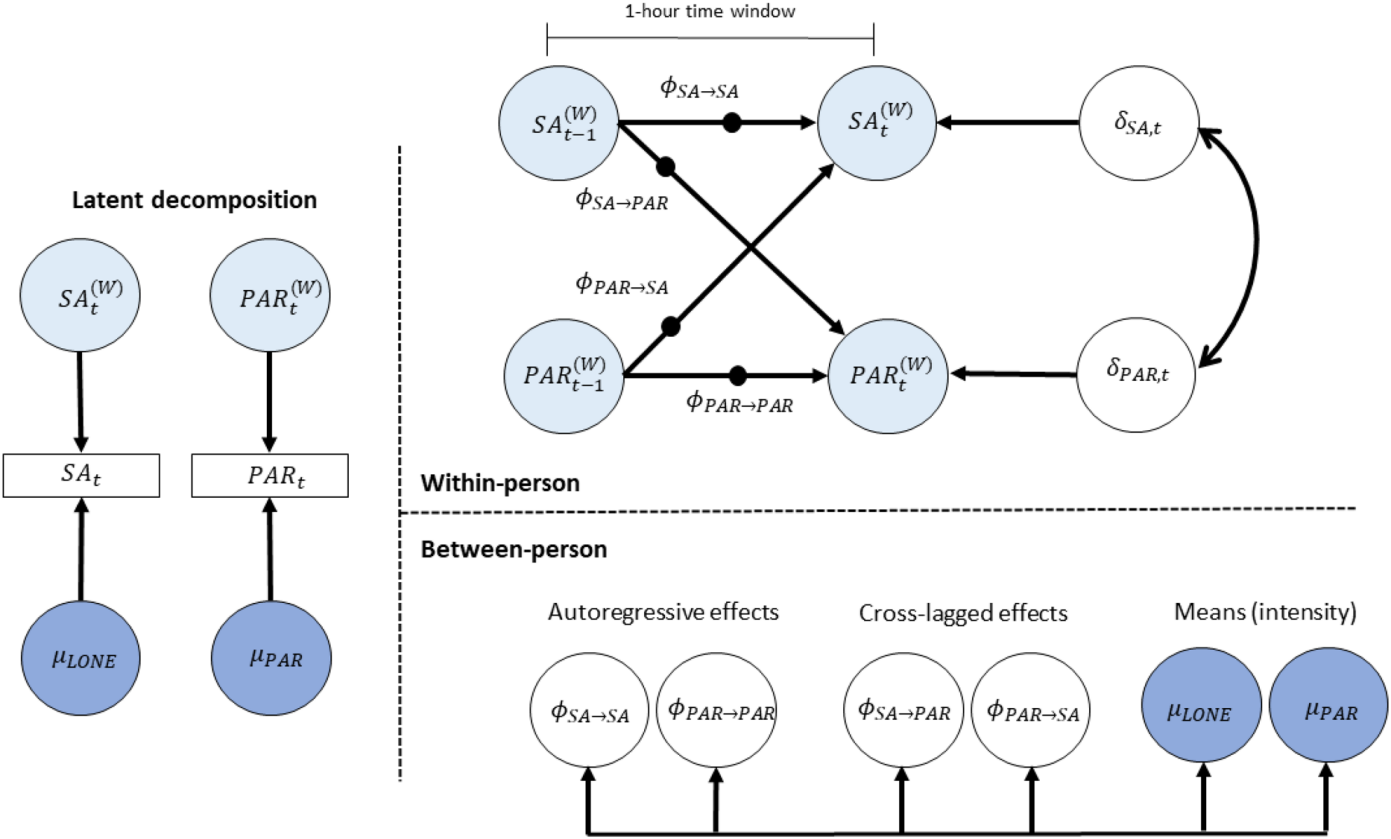
Schematic representation of the DSEM model of social anxiety and paranoia (Model 1). *Note*: This figure is a schematic representation of the dynamic structural equation model of social anxiety and paranoia (Model 1). The left panel contains the decomposition of social anxiety and paranoia into within-person and between-person variance components respectively. The top right panel indicates the within-person level model, which is a vector autoregressive model. The bottom right panel indicates the between-person level model, which includes all the random effects of the model, corresponding to the solid black circles in the within-person level model. SA—social anxiety, PAR—paranoia.

**Figure 2:**
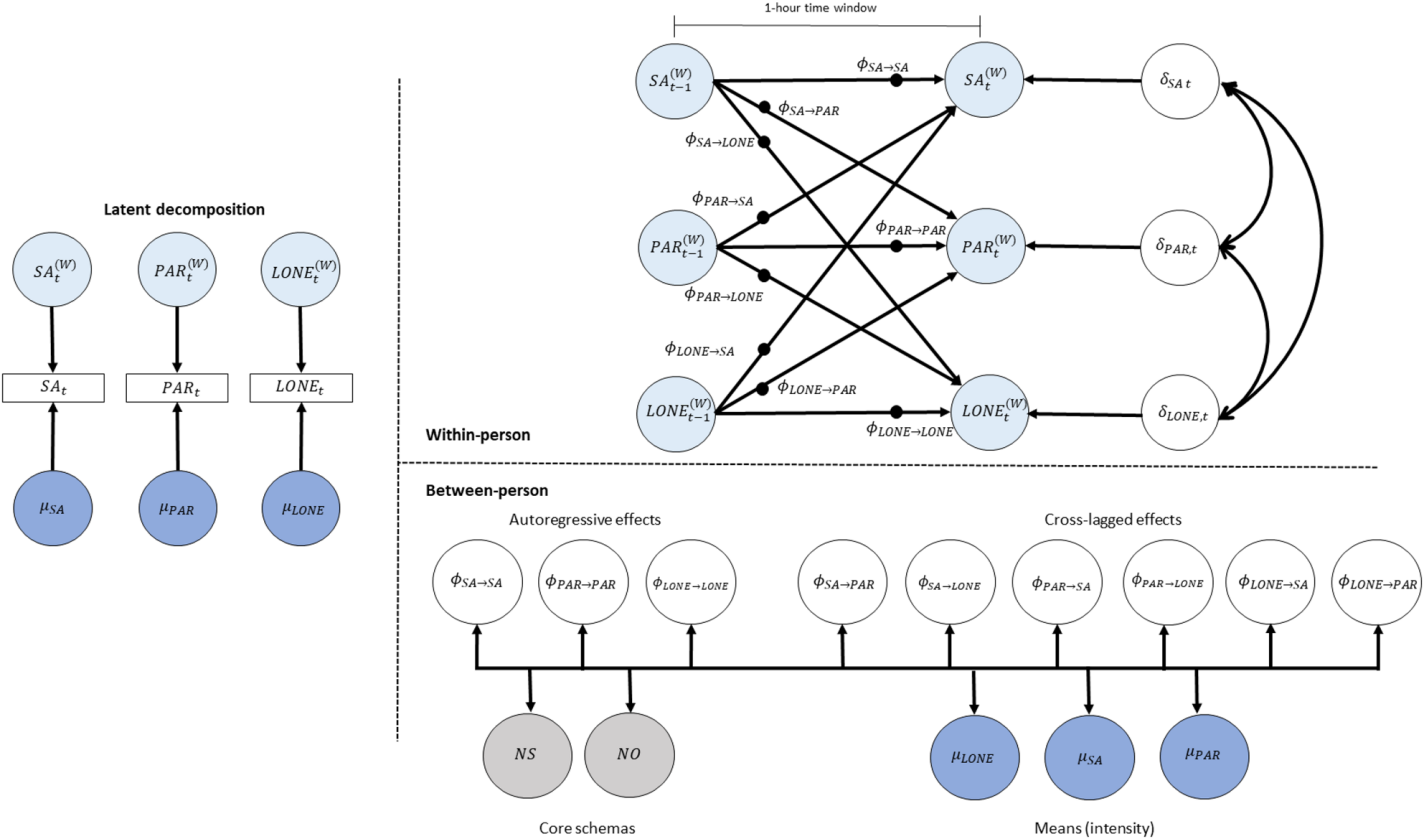
Schematic representation of the DSEM model of loneliness, social anxiety and paranoia (Model 2). *Note*: This figure is a schematic representation of the dynamic structural equation model of social anxiety, paranoia and loneliness (Model 2). The left panel contains the decomposition of social anxiety, paranoia and loneliness into within-person and between-person variance components respectively. The top right panel indicates the within-person level model, which is a vector autoregressive model. The bottom right panel indicates the between-person level model, which includes the levels of negative-self and -other schemas, as well as all the random effects of the model, corresponding to the solid black circles in the within-person level model. SA—social anxiety, PAR—paranoia, LONE—loneliness, NS—negative-self subscore of the Brief Core Schema Scale, NO—negative-other subscore of the Brief Core Schema Scale.

For the first hypothesis, we fitted the bivariate multilevel first-order vector autoregressive model (Model 1) with within-person cross-lagged effects between momentary social anxiety and paranoia, while controlling for their autoregressive effects (see schematic representation in Fig. 1). For the second hypothesis, we further added momentary loneliness into the model, creating a Model 2 that examined the within-person cross-lagged effects between loneliness, social anxiety and paranoia, while controlling for their autoregressive effects (see Fig. 2). As exploratory analyses, we also examined gender differences in these effects by adding gender as a predictor at the between-person level of these models. The third hypothesis was tested by the correlation between the random effects at the between-person level and the levels of negative-self and -other schemas, which were grand-mean centred before entering into the model.

## Data Availability

The datasets generated during and/or analyzed during the current study are available from the corresponding author upon reasonable request.
